# Technical and anatomical factors affecting the size of the branch pulmonary arteries following first stage Norwood palliation for HLHS

**DOI:** 10.1186/1532-429X-17-S1-Q88

**Published:** 2015-02-03

**Authors:** Sophie Bertaud, Tarique Hussain, Gerald F Greil, Mohamed S Nassar

**Affiliations:** 1 Division of Imaging Sciences & Biomedical Engineering, King's College London, London, UK; 2 Paediatric Cardiology, Evelina London Children's Hospital, Guy's & St. Thomas' NHS Foundation Trust, London, UK; 3 Cardiothoracic Surgery, Evelina London Children's Hospital, Guy's & St. Thomas' Hospital, London, UK

## Background

Evidence suggests that the anatomical characteristics of the Fontan circulation, in particular the size of the pulmonary arteries, play a key role in the hemodynamic efficiency of the circulation and consequently, patient outcome. Understanding and optimizing early growth of the pulmonary arteries is crucial to avoid later increase in pulmonary vascular resistance, a major risk factor for failure of the Fontan circulation. We sought to elucidate the factors that may account for central pulmonary artery stenosis and a small left pulmonary artery (LPA) following the first Norwood operation for Hypoplastic Left Heart Syndrome (HLHS).

## Methods

47 consecutive patients, underwent cardiac MRI prior to hemifontan. Measurements were taken using first-pass 3-dimensional contrast-enhanced angiography. Factors investigated included: size and site of the Pulmonary Artery (PA) bifurcation stump in relation to the Damus-Kaye-Stansel anastomosis (DKS), inter-aortic distance (*distance between the neo aorta and the descending aorta*, *IAD*), inter-aortic ratio (IAD/antero-posterior dimension of the chest), distance from the LPA to the under surface of the aortic arch, the size of the native aorta and PA, and the type of arch reconstruction technique.

## Results

Stenosis occurred either centrally, at the origin of the branch pulmonary artery (BPA), or more distally, in the mid left pulmonary artery (LPA) (posterior to the DKS). There was a significantly lower incidence of central BPA stenosis when the confluence was placed in the mid position compared to right/left position (26% vs 67%, p=0.011). A more bulky confluence was also found in those patients with central branch PA stenosis (186 vs 137 mm^2^/m^2^; p=0.047). The mid-LPA consistently showed antero-posterior compression (mean cranio-caudal diameter 3.82mm vs mean antero-posterior diameter 3.07mm, p<0.001). Indexed mid-LPA area was only correlated with IAD/IAR (r= 0.49 and 0.51, P<0.001). No correlation was shown with the distance to the under surface of the arch (r=0.14, p=0.37), again confirming antero-posterior compression of the LPA rather than cranio-caudal. In multivariable analysis, the only predictor of indexed mid LPA area was the IAR (P<0.001). There was no significant difference in the IAD or IAR between the two arch reconstruction techniques (mean IAD 15.5 vs 13.5mm (p=0.14)); (mean IAR 0.17 vs 0.19 (p=0.21)).

## Conclusions

Our study highlights IAR and the size and position of the PA confluence as playing a key role in LPA growth and central BPA stenosis. These anatomical variations and technical considerations should be taken into account during surgery and when counseling for long-term outcomes in HLHS. This study demonstrates how imaging feedback and surgical techniques could be used in an iterative manner aiming to constantly improve outcome.

## Funding

N/A.

**Figure 1:**
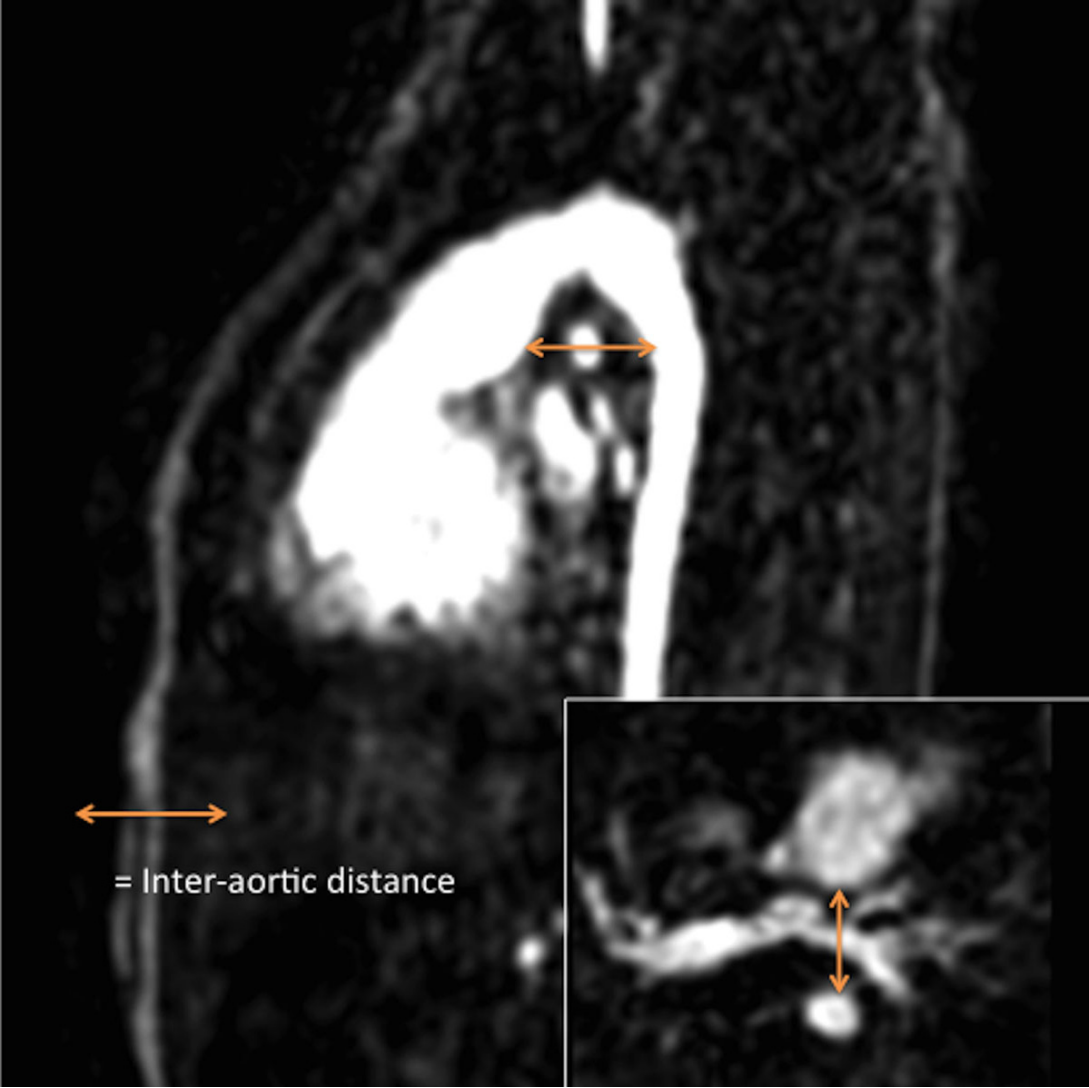
Measurement of Inter-Aortic Distance (IAD) on a sagittal and an axial view.

**Figure 2:**
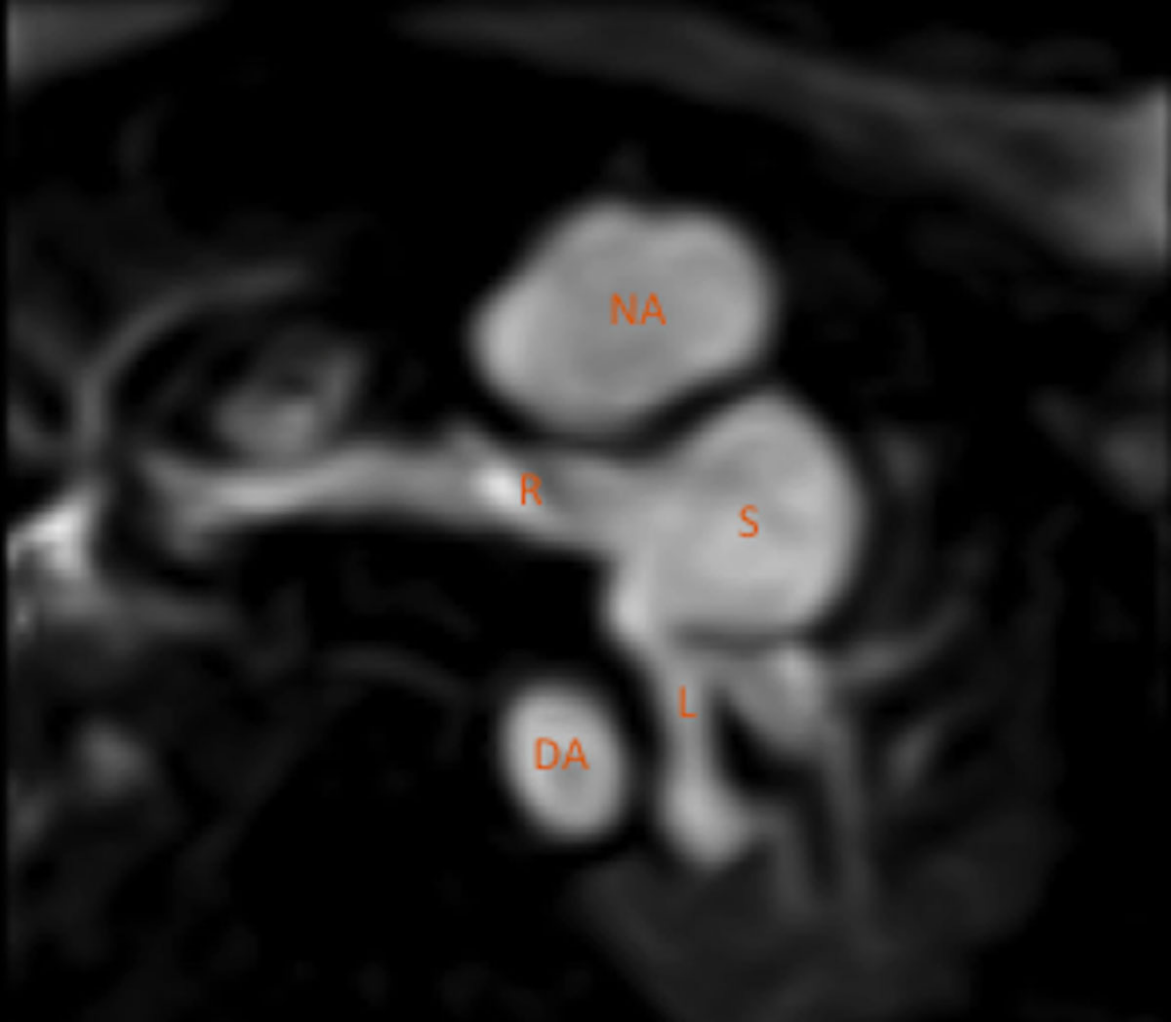
MRI Gadolinium enhanced image showing the PA confluence and stump positioned to the left of the DKS with LPA origin kinking. NA : Neo-Aorta. S: Stump. R: Right pulmonary artery. L: Left pulmonary artery. DA: Descending Aorta

